# The effectiveness of oral anti-SARS-CoV-2 agents in non-hospitalized COVID-19 patients with nonalcoholic fatty liver disease: a retrospective study

**DOI:** 10.3389/fphar.2024.1321155

**Published:** 2024-02-15

**Authors:** Chun-Chi Yang, Ya-Wen Tsai, Su-Hung Wang, Jheng-Yan Wu, Ting-Hui Liu, Wan-Hsuan Hsu, Po-Yu Huang, Min-Hsiang Chuang, Ming-Jen Sheu, Chih-Cheng Lai

**Affiliations:** ^1^ Division of Hepato-Gastroenterology, Department of Internal Medicine, Chi Mei Medical Center, Tainan, Taiwan; ^2^ Center for Integrative Medicine, Chi Mei Medical Center, Tainan, Taiwan; ^3^ Department of Medical Laboratory Sciences and Biotechnology, Fooyin University, Kaohsiung, Taiwan; ^4^ Department of Nutrition, Chi Mei Medical Center, Tainan, Taiwan; ^5^ Department of Psychiatry, Chi Mei Medical Center, Tainan, Taiwan; ^6^ Department of Internal Medicine, Chi Mei Medical Center, Tainan, Taiwan; ^7^ Division of Hospital Medicine, Department of Internal Medicine, Chi Mei Medical Center, Tainan, Taiwan; ^8^ School of Medicine, College of Medicine, National Sun Yat-sen University, Kaohsiung, Taiwan

**Keywords:** COVID-19, molnupiravir, nirmatrelvir plus ritonavir, nonalcoholic fatty liver disease, outcome

## Abstract

**Background:** The effectiveness of the novel oral antiviral agents, nirmatrelvir plus ritonavir and molnupiravir, in treating COVID-19 in patients with nonalcoholic fatty liver disease is unclear.

**Objective:** To assess the effectiveness of novel oral antiviral agents against COVID-19 among patients with nonalcoholic fatty liver diseases.

**Methods:** This retrospective cohort study used the TriNetX Research Network to identify non-hospitalized patients with COVID-19 and nonalcoholic fatty liver disease between 1 January 2022, and 30 June 2023. Propensity score matching was used to form two matched cohorts treated with or without nirmatrelvir-ritonavir or molnupiravir.

**Results:** In the two matched cohorts of 6,358 patients each, the use of novel oral antiviral agents was associated with a significantly lower risk of all-cause emergency department visits, hospitalization, or mortality (6.59% versus 8.24%; hazard ratio [HR], 0.80; 95% confidence interval [CI], 0.70–0.91). The novel antiviral group had a significantly lower risk of all-cause emergency department visits (HR, 0.85; 95% CI, 0.74–0.99). Additionally, the incidence of hospitalization was significantly lower in the oral antiviral group than in the control group (HR, 0.71; 95% CI, 0.55–0.90). There were no deaths in the oral antiviral group but 12 deaths in the control group.

**Conclusion:** Novel oral antiviral agents are beneficial for treating COVID-19 in patients with nonalcoholic fatty liver disease.

## Introduction

Nonalcoholic fatty liver disease (NAFLD) is a prevalent chronic liver disease worldwide. According to the Global Burden of Diseases, Injuries, and Risk Factors Study, it affects approximately 15,023 individuals per 100,000 population, with an annual increase of 0.83% from 2000 to 2019 (Younossi et al., 2011; Marjot et al., 2021; Chew et al., 2023). The US National Health and Nutrition Examination Surveys have shown a gradual increase in prevalence in the US, from 20.0% in 1988–1994, to 28.3% in 1999–2004, 33.2% in 2009–2012, and 31.9% in 2013–2016 (Younossi et al., 2020). A meta-analysis showed the highest prevalence in the Middle East (37.19%) and South America (30.45%), followed by Asia (27.17%), North America (24.13%), Europe (23.17%), and Africa (13.48%) (Younossi et al., 2016), suggesting that the burden of NAFLD is high worldwide.

Recently, several studies have shown that NAFLD and other types of chronic liver disease, including alcohol-related liver disease, autoimmune hepatitis, and cirrhosis, may increase the risk of severe coronavirus disease (COVID-19) (Singh and Khan, 2020; Marjot et al., 2021; Middleton et al., 2021; Vardavas et al., 2022). A retrospective study conducted in China found that patients with NAFLD had a higher risk of COVID-19 progression (odds ratio [OR], 6.4; 95% confidence interval [CI], 1.5–31.2) and longer viral shedding time (17.5 ± 5.2 days vs. 12.1 ± 4.4 days, *p* < 0.0001) compared with patients without NAFLD (Ji et al., 2020). Another study found that patients with NAFLD and SARS-CoV-2 infection were more likely to present with severe illness on admission and used more hospital resources (Younossi et al., 2022). Moreover, Rui et al. (Huang et al., 2020) found that patients with NAFLD had an increased risk of developing liver injury if they developed COVID-19 (OR, 2.956; 95% CI, 1.526–5.726; *p* = 0.001). Lastly, a systematic review and meta-analysis conducted in 2021 revealed that patients with NAFLD had an increased risk of severe COVID-19 (adjusted odds ratio [aOR], 2.60; 95% CI, 2.24–3.06; *p* < 0.001) and intensive care unit (ICU) admission (aOR, 2.60; 95% CI, 1.26–2.60; *p* < 0.001) (Singh et al., 2021). Because NAFLD is associated with severe COVID-19, effective management of COVID-19 in this high-risk population is important. Several oral antiviral agents such as nirmatrelvir plus ritonavir (NMV-r, Paxlovid™) and molnupiravir (MOV) are recommended for non-hospitalized high-risk patients with COVID-19 (Bhimraj et al., 2022). Paxlovid, consists of nirmatrelvir, which is boosted by the addition of ritonavir. The active ingredient nirmatrelvir works by reducing the ability of SARS-CoV-2 to multiply within the body through a mechanism of inhibition of some viral proteases necessary for the virus to replicate. Ritonavir, on the other hand, inhibits the metabolism operated by cytochrome P450 prolonging the action of nirmatrelvir, allowing to remain within the body in quantities that allow it to interfere with the replication of the virus for a longer period of time than would happen if the nirmatrelvir was administered alone. However, there is a paucity of evidence regarding the effectiveness of these drugs in patients with NAFLD. Therefore, we conducted a multicenter retrospective study to investigate the short-term clinical effectiveness of these oral antiviral agents in patients with NAFLD and COVID-19.

## Materials and methods

### Data source

Patient data were retrieved from the TriNetX Research Network, a global health collaborative clinical research platform containing deidentified records of more than 100 million patients. This research network includes de-identified electronic medical records from multiple healthcare organizations worldwide. The records contain ICD-10 diagnoses, procedures, medications, laboratory data, and genomic information. This study was approved by the Institutional Review Board of Chi Medical Center (No. 11202-002) and the requirement for informed consent was waived because of its retrospective design.

### Patient selection

This retrospective study analyzed data of adult patients (aged ≥18 years), with more than two visits to a healthcare provider, who had preexisting NAFLD diagnosed at least 3 months prior, and either tested positive for SARS-CoV-2 infection or were diagnosed with COVID-19 between 1 January 2022, and 30 June 2023. Patients with COVID-19 were identified using ICD-10 diagnosis codes (U07.1, J12.81, and J12.82), positive SARS-CoV-2 RNA tests (LOINC 94309-2, 94,500-6, 95,406-5, 94,502-2, 94,565-9, 95,608-6, 94,759-8, and 94,845-5), and SARS-CoV-2 antigen immunoassays (LOINC 94558-4 and 96,119-3). Patients with NAFLD were identified using the ICD-10 codes K76.0 (fatty liver, not elsewhere classified) and K75.81 (nonalcoholic steatohepatitis).

As the TriNetX database does not provide information on the severity of COVID-19, and considering that oral antivirals were specifically recommended for patients with mild-to-moderate cases, we focused our study exclusively on non-hospitalized patients to ensure both the study and control groups fell within this category. Patients were excluded from the analysis if they were admitted to hospital from 3 days before to 1 day after the COVID-19 diagnosis; received convalescent plasma or monoclonal antibody treatment (tixagevimab, cilgavimab, bebtelovimab, and bamlanivimab); died on the day of COVID-19 diagnosis; had been diagnosed with chronic liver disease not related to NAFLD; had a history of excessive alcohol use/abuse or alcohol-related disorders; or had both NAFLD and other coexisting liver diseases. Supplementary Table S1 provides additional information on the inclusion and exclusion criteria.

The patients were further divided into those who received oral antivirals (NMV-r or MOV) within 5 days of the COVID-19 diagnosis (oral antiviral group) and untreated patients (control group).

### Covariates

Propensity score matching was performed in a 1:1 ratio using the TriNetX built-in platform. Demographic characteristics (age, sex, and race) and comorbidities (asthma [45], diabetes mellitus [ICD10 code E08-E13], hypertension [ICD10 code E10], ischemic heart disease [I20–I25], respiratory diseases [J00–J99], chronic kidney disease [N18], chronic lower respiratory diseases [J40–J47], and neoplasms [C00–D49]) were used to eliminate the confounding factors and selection bias. A standard difference of <0.1 was considered an acceptable balanced match.

### Outcomes

Primary outcome was defined as the composite outcome of all-cause emergency department (ED) visits, hospitalization, or mortality during the 30-day follow-up period. Second outcomes were the individual outcomes of all-cause ED visits, hospitalization, and mortality during the follow-up.

### Statistical analyses

Statistical analyses were performed using the TriNetX platform, and baseline characteristics were reported as means and standard deviations, or frequencies and proportions. Hazard ratios (HRs) with 95% confidence intervals (CIs) were calculated using Cox proportional hazards regression. The cumulative probability was determined using Kaplan–Meier curves, with statistical significance set at *p* < 0.05. Subgroup analyses were conducted based on patient age, sex, and hypertension, diabetes, dyslipidemia, obesity, vaccination status and type of oral antivirals.

## Results

**FIGURE 1:**
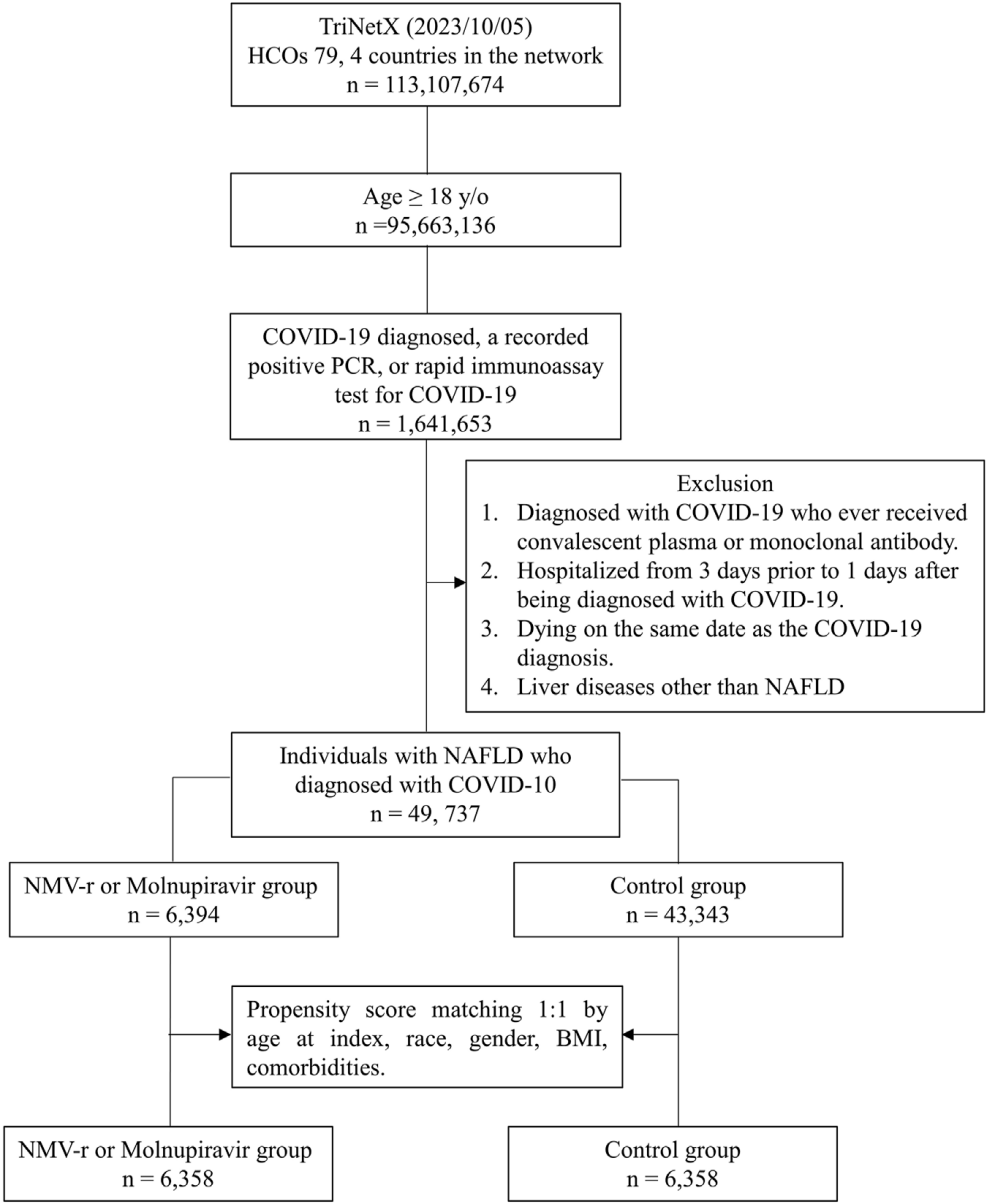
Flowchart of patient selection and the cohort selection process.

Based on the initial database search, a total of 49,737 patients with NAFLD and COVID-19 were identified. Of these, 6,394 patients belonged to the oral antiviral group, while 43,343 patients were in the control group (Figure 1). Table 1 displays the baseline characteristics of the patients included in the analysis before and after propensity score matching. Before matching, the oral antiviral group was characterized by older age and a higher proportion of patients classified as white race compared to the control group. Additionally, the oral antiviral group exhibited a higher prevalence of systemic disorders, including endocrine, nutritional, and metabolic diseases, diseases of the circulatory and nervous systems, as well as skin and subcutaneous diseases, in comparison to the control group. Specifically, the oral antiviral group had a greater prevalence of essential hypertension, hyperlipidemia, and neoplasms than the control group. After propensity score matching, two matched cohorts, each consisting of 6,358 patients, were identified, resulting in a total of 12,716 patients included in the analysis. The absolute standardized mean differences between the study and control groups were all <0.1, indicating a successful match between the two groups.

**TABLE 1:** Baseline characteristics of antiviral and non-antiviral group in patients with nonalcoholic fatty liver disease and COVID-19 before and after propensity score mathcing matching.

	Before matching			After matching		
	Oral antiviral group (n = 6,358)	Control group (n = 43,215)	Std diff	Oral antiviral group (n = 6,358)	Control group (n = 6,358)	Std diff
Age at index, years	58.2 ± 13.7	55.5 ± 15.0	0.185	58.2 ± 13.7	58.3 ± 14.2	0.011
Sex						
Male	2,365 (37.2%)	15,957 (36.9%)	0.006	2,365 (37.2%)	2,362 (37.2%)	0.001
Female	3,639 (57.2%)	26,360 (61.0%)	0.077	3,639 (57.2%)	3,668 (57.7%)	0.009
Race						
White	4,930 (77.5%)	30,598 (70.8%)	0.154	4,930 (77.5%)	4,954 (77.9%)	0.009
Black or African American	359 (5.6%)	3,879 (9.0%)	0.128	359 (5.6%)	358 (5.6%)	0.001
Asian	220 (3.5%)	2,062 (4.8%)	0.066	220 (3.5%)	223 (3.5%)	0.003
Unknown Race	808 (12.7%)	6,242 (14.4%)	0.051	808 (12.7%)	776 (12.2%)	0.015
Organ system dysfunction						
Endocrine, nutritional, and metabolic diseases	5,849 (92.0%)	38,008 (88.0%)	0.135	5,849 (92.0%)	5,856 (92.1%)	0.004
Diseases of the circulatory system	4,883 (76.8%)	31,992 (74.0%)	0.064	4,883 (76.8%)	4,949 (77.8%)	0.025
Diseases of the nervous system	4,581 (72.1%)	28,904 (66.9%)	0.112	4,581 (72.1%)	4,432 (69.7%)	0.052
Diseases of the respiratory system	4,253 (66.9%)	28,695 (66.4%)	0.010	4,253 (66.9%)	4,227 (66.5%)	0.009
Diseases of the genitourinary system	4,098 (64.5%)	27,864 (64.5%)	<0.001	4,098 (64.5%)	4,126 (64.9%)	0.009
Diseases of the skin and subcutaneous tissue	3,369 (53.0%)	20,441 (47.3%)	0.114	3,369 (53.0%)	3,442 (54.1%)	0.023
Underlying diseases						
Essential hypertension	4,196 (66.0%)	27,152 (62.8%)	0.066	4,196 (66.0%)	4,282 (67.3%)	0.029
Hyperlipidemia	2,967 (46.7%)	19,397 (44.9%)	0.036	2,967 (46.7%)	3,026 (47.6%)	0.019
Neoplasms	2,628 (41.3%)	16,022 (37.1%)	0.087	2,628 (41.3%)	2,658 (41.8%)	0.010
Diabetes mellitus	2,481 (39.0%)	16,937 (39.2%)	0.003	2,481 (39.0%)	2,470 (38.8%)	0.004
Obesity	2,153 (33.9%)	15,005 (34.7%)	0.018	2,153 (33.9%)	2,139 (33.6%)	0.005
Chronic lower respiratory diseases	1,938 (30.5%)	13,066 (30.2%)	0.005	1,938 (30.5%)	1,899 (29.9%)	0.013
Asthma	1,324 (20.8%)	8,826 (20.4%)	0.010	1,324 (20.8)	1,298 (20.4%)	0.010
Ischemic heart diseases	1,019 (16.0%)	8,181 (18.9%)	0.077	1,019 (16.0%)	1,060 (16.7%)	0.017
Chronic kidney disease	622 (9.8%)	5,202 (12.0%)	0.072	622 (9.8%)	627 (9.9%)	0.003

Standardized difference (Std diff) < 0.1 is considered an insignificant difference.

### Primary outcome

**FIGURE 2:**
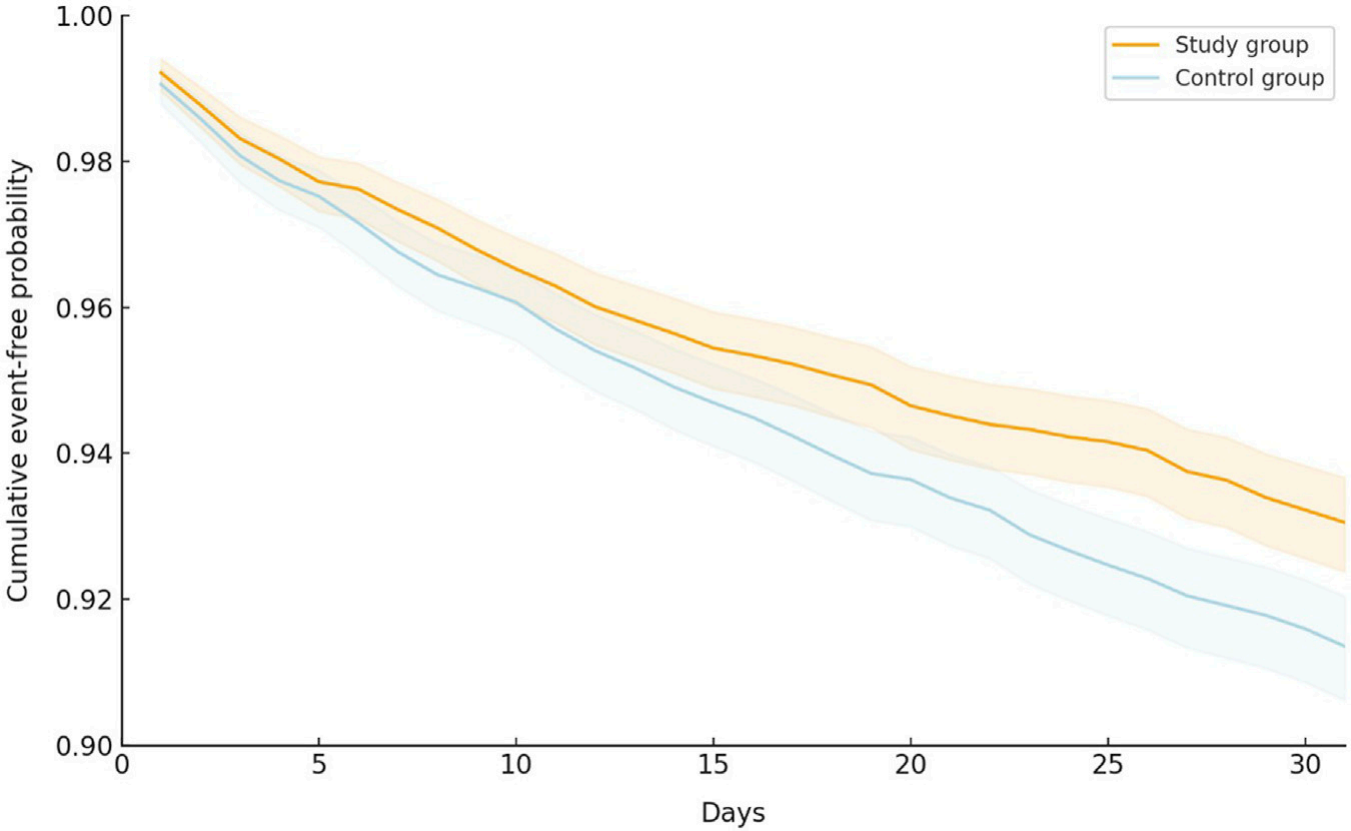
Kaplan–Meier curves of the primary outcome (composite of all-cause emergency department visits, hospitalization, or mortality).

During the 30-day follow-up period, the oral antiviral group had a significantly lower incidence of all-cause ED visits, hospitalization, or mortality than the control group, with 419 (6.59%) patients in the oral antiviral group and 524 (8.24%) patients in the control group experiencing the composite outcome of all-cause ED visits, hospitalization, or mortality (HR, 0.80; 95% CI, 0.70–0.91) (Table 2). The Kaplan–Meier curves of the probability of time-to-event, showed that the risk of all-cause ED visits, hospitalization, or mortality was significantly lower in the oral antiviral group than control group (*p* < 0.001, log-rank test) (Figure 2).

**TABLE 2:** The hazard ratio for comparing matched nirmatrelvir-ritonavir or molnupiravir between control cohorts for the outcomes.

Outcomes	Patients with outcome		Hazard ratio (95%CI)	*p*-value
	Antiviral group (n = 6,358)	Control group (n = 6,358)		
Primary outcome				
All-cause ED visits, hospitalization, or death	419	524	0.80 (0.70–0.91)	<0.001
Secondary outcome				
All-cause ED visits	329	386	0.85 (0.74–0.99)	0.03
All-cause hospitalization	112	159	0.71 (0.55–0.90)	0.05
All-cause death	0	12	-	-

### Secondary outcomes

Regarding individual outcomes, the oral antiviral group had a significantly lower risk of all-cause ED visits than the control group (5.17% vs. 6.07%; HR, 0.85; 95% CI, 0.74–0.99). The incidence of all-cause hospitalization was also lower in the oral antiviral group than that in the control group (HR, 0.71; 95% CI, 0.55–0.90). There were no deaths in the oral antiviral group but 12 deaths in the control group during the follow-up period (Table 2).

### Subgroup analyses

**FIGURE 3:**
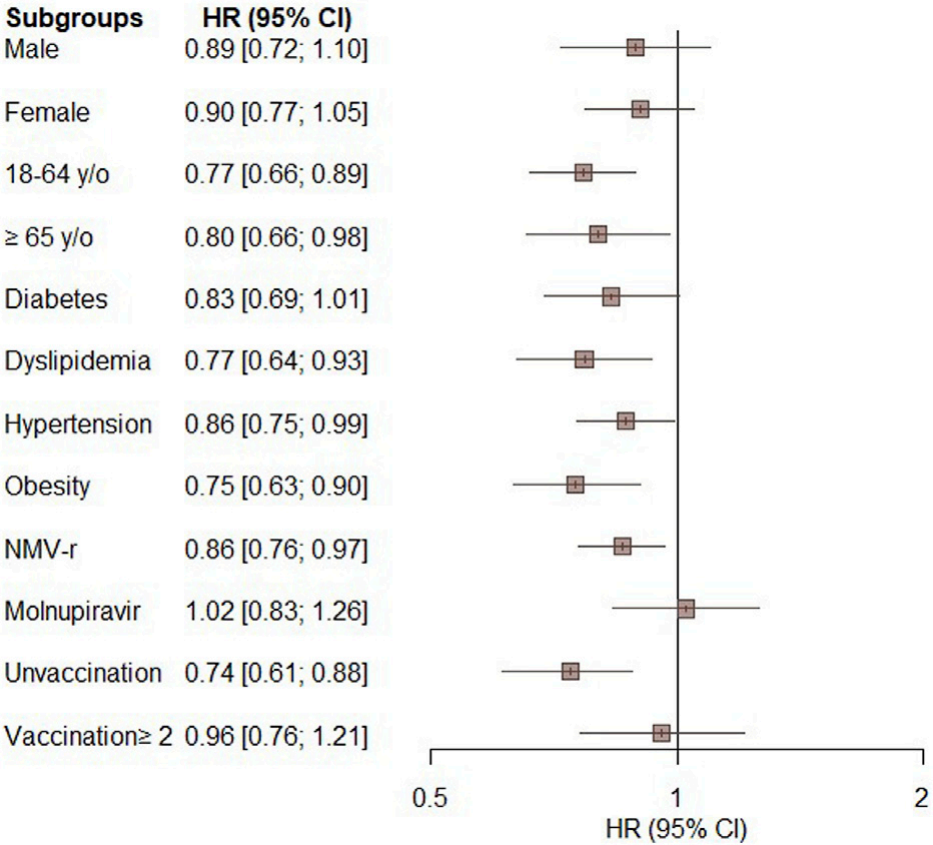
Subgroup analysis of the primary outcome (composite of all-cause emergency department visits, hospitalization, or mortality) in patients treated with novel oral antiviral agents compared with untreated patients.

Figure 3 displays the results of the subgroup analyses. The significantly lower risk of all-cause ED visits, hospitalization, or mortality in the oral antiviral group compared to the control group was consistent across most of subgroups, including patients aged 18–64 years (HR, 0.77; 95% CI, 0.66–0.89) and those aged ≥65 years (HR, 0.80; 95% CI, 0.66–0.98), patients with dyslipidemia (HR, 0.77; 95% CI, 0.64–0.93), hypertension (HR, 0.87; 95% CI, 0.75–0.99), obesity (HR, 0.75; 95% CI, 0.63–0.90), patients receiving NMV-r (HR, 0.86; 95% CI, 0.76–0.97), and unvaccinated patients (HR, 0.74; 95% CI, 0.61–0.88). Additionally, there was a non-significant trend towards a lower risk of the primary outcome in subgroups comprising males, females, individuals with diabetes, those receiving molnupiravir, and those who had received more than 2 vaccinations (Figure 3).

## Discussion

To our knowledge, this retrospective study of 12,716 patients is the first to evaluate the short-term effects of novel oral antiviral agents for the treatment of COVID-19 in patients with NAFLD. Our findings suggest that treatment with oral antiviral agents can improve short-term outcomes in this patient population, as evidenced by the significantly lower risk of all-cause ED visits, hospitalization, and mortality in patients receiving oral antiviral agents than in the untreated control group. This lower risk of primary outcomes was consistent across most of subgroups based on age, sex, and underlying comorbidities and the type of antiviral agents despite some differences did not reach statistical significances. The oral antiviral group also had a significantly lower risk of all-cause ED visits and hospitalization than the control group. Lastly, there were no deaths in the oral antiviral group compared with 12 deaths in the control group. These findings suggest that novel oral antiviral agents have a beneficial effect in the treatment of COVID-19 in patients with NAFLD and support their use in this vulnerable population.

Similar to previous studies (Li et al., 2021; Li et al., 2022; Ahmed et al., 2023), we found that patients with NAFLD had a high prevalence of cardio-metabolic dysfunction and associated diseases (Table 1). Diabetes mellitus has been described as an additional risk factor for severe COVID-19 (Guo et al., 2020). Independent of diabetes, the presence of adiposity (i.e., excess body fat that impairs health, including overweight, obesity, and metabolic syndrome) appears to be a risk factor in infectious viral diseases due to impaired immunity. Obese individuals with COVID-19 are at higher risk of hospitalization, ICU admission, and mortality (Popkin et al., 2020). Based on the subgroup analysis, the clinical benefit of novel oral antiviral agents remained in the specific subgroup of patients with dyslipidemia, hypertension, obesity or diabetes mellitus, which is consistent with the present COVID-19 treatment guidelines (Bhimraj et al., 2022).

Metabolic dysfunction-associated fatty liver disease (MAFLD) is a new term proposed in 2020 that includes hepatic steatosis along with the presence of at least one of the following conditions: obesity, type 2 diabetes, or evidence of multiple metabolic risk abnormalities (Eslam and George, 2019). Unlike NAFLD, MAFLD is more closely associated with the main risk factors of atherosclerosis and cardiovascular disease (Boccatonda et al., 2023). The MAFLD definition has higher sensitivity for detecting significant fibrosis (Yamamura et al., 2020). In a US population-based study, MAFLD was associated with an increased risk of all-cause mortality, whereas NAFLD was not associated with all-cause mortality after adjusting for metabolic risk factors (Kim et al., 2021). Furthermore, many studies (Zhou et al., 2020a; Zhou et al., 2020b; Gao et al., 2021) and a meta-analysis of 16 studies with a total of 11,484 patients found that MAFLD was associated with an increased risk of severe COVID-19 (OR, 3.07; 95% CI, 2.30–4.09), and ICU admission (OR, 1.46; 95% CI, 1.12–1.91) (Hayat et al., 2022). In this study, the subgroup analysis showed that the administration of novel oral antiviral agents might provide potential clinical benefits for patients with NAFLD and comorbidities such as obesity, dyslipidemia, and hypertension, which are considered to be associated with MAFLD. Although we may not have captured all patients with MAFLD based on the TriNetX platform, we were able to identify a subset of patients with NAFLD and coexisting major metabolic risk abnormalities similar to MAFLD, who may potentially have similar outcomes in the context of COVID-19. Accordingly, our findings suggest the use of novel oral antiviral agents is effective for treating COVID-19 in patients with MAFLD.

The choice of antiviral agent should consider various factors, including patient characteristics, comorbidities, drug interactions, and local availability. Previous studies have suggested that NMV-r may be more effective than MOV in treating patients with COVID-19 (Lai et al., 2022). Similarly, this study found that patients receiving NMV-r had better outcomes than untreated patients, but the beneficial effect of MOV was not statistically significant. This finding aligns with current guidelines, which recommend using MOV in situations where other antiviral agents are not available, feasible, or clinically appropriate (National Institutes of Health, 2023).

This study had several strengths. First, although chronic liver disease is known to be an indication for the use of an intensive antiviral agent, to our knowledge, this is the first study to focus on the effectiveness of novel antiviral agents for treating COVID-19 in patients with NAFLD. Second, we included robust controls and adjusted for baseline characteristics and confounders using a large sample in the propensity-matched analyses, resulting in narrow CIs. Third, we performed a subgroup analysis including hypertension, dyslipidemia, diabetes, and obesity, which helps to assess the effectiveness of antiviral agents for treating COVID-19 in patients with MAFLD. Finally, our results remained consistent across sensitivity analyses of risk factors for mortality.

This study also has some limitations. The patient data, derived from an electronic health record-based database, were susceptible to errors in diagnostic coding and data entry. In addition, coding errors may have been present. Because NAFLD is an exclusive diagnosis, we considered all diagnostic codes for liver disease that should be excluded according to the NAFLD diagnostic criteria in as much detail as possible. However, there may have been errors in diagnosis. Second, despite our efforts to adjust for many confounding factors, there is still a possibility of residual confounding. Our data did not include imaging modalities, such as conventional imaging techniques and elastography, to confirm the diagnosis of NAFLD. We could not evaluate the severity and progressive forms of NAFLD, such as nonalcoholic steatohepatitis and cirrhosis, owing to the unavailability of common surrogate serum marker-based fibrosis scoring systems, including the NAFLD fibrosis score, Fibrosis-4 Index, and ALT/AST ratio. Finally, our inclusion criteria were not based on a histological diagnosis, which may have increased the risk of misdiagnosis. Further studies are warranted to confirm these findings.

In conclusion, this retrospective study found that treatment with novel oral antiviral agents within 5 days of COVID-19 diagnosis was associated with a lower risk of all-cause ED visits, hospitalization, and mortality in patients with preexisting NAFLD. Therefore, early treatment with novel oral antiviral agents may be beneficial in patients with NAFLD and COVID-19. In subgroup analysis, patients with NAFLD and major metabolic disorders still had significantly better outcomes with the use of oral antiviral agents. These findings suggest the potential role of the antiviral agents in patients with MAFLD. Further studies are needed to confirm these findings and investigate the long-term outcomes of this treatment approach.

## Data Availability

The original contributions presented in the study are included in the article/Supplementary Material, further inquiries can be directed to the corresponding authors.
