# Supplementary material for: The effectiveness of oral anti-SARS-CoV-2 agents in non-hospitalized COVID-19 patients with nonalcoholic fatty liver disease: a retrospective study
Source: Front Pharmacol. 2024 Feb 15;15:1321155. doi: 10.3389/fphar.2024.1321155 (PMC10902026; doi:10.3389/fphar.2024.1321155)
Supplement: Supplementary file 1 [file Table1.docx]

**Supplementary methods**

# Introduction

TriNetX is a global platform that provides access to electronic health records containing information such as diagnoses, procedures, medications, laboratory results, and genomic data from significant healthcare institutions. The analysis was conducted on the "research network" within the TriNetX platform, which includes 76 healthcare organizations.

# Methods

The analysis process involves two main steps: 1) Defining cohorts based on query criteria; and 2) Setting up the index event, outcomes criteria, and the time frame for analysis. The Propensity Score Matching section includes characteristics of the cohorts that were balanced using propensity score matching.

## Cohorts definition

This section lists all terms used in the definitions of the two cohorts.

### **Table S1**

This query was run on the network Research with 76 HCO(s) queried and 76 HCO(s) responded. A total of 25 provider(s) responded with patients. The final cohort included 3,794 patients who matched the query criteria listed in the table below.

|  | Inclusion criteria | Exclusion criteria |
| --- | --- | --- |
| Patients with COVID-19  (Jan 1, 2021 and Dec 31, 2022) | - U07.1 COVID-19, - J12.81 Pneumonia due to SARS-associated coronavirus, - J12.82 Pneumonia due to coronavirus disease 2019, - TNX:9088 SARS coronavirus 2 and related RNA [Presence] (labResult: Positive) - 94309-2 SARS-CoV-2 (COVID-19) RNA [Presence] in Specimen by NAA with probe - 94500-6 SARS-CoV-2 (COVID-19) RNA [Presence] in Respiratory specimen by NAA with probe detection (labResult: Positive) - 94502-2 SARS-related coronavirus RNA [Presence] in Respiratory specimen by NAA with probe detection (labResult: Positive) - 95406-5 SARS-CoV-2 (COVID-19) RNA [Presence] in Nose by NAA with probe detection (labResult: Positive) - 94565-9 SARS-CoV-2 (COVID-19) RNA [Presence] in Nasopharynx by NAA with non-probe detection (labResult: Positive) - 95608-6 SARS-CoV-2 (COVID-19) RNA [Presence] in Respiratory specimen by NAA with non-probe detection (labResult: Positive) - 94759-8 SARS-CoV-2 (COVID-19) RNA [Presence] in Nasopharynx by NAA with probe detection (labResult: Positive) - 94845-5 SARS-CoV-2 (COVID-19) RNA [Presence] in Saliva (oral fluid) by NAA with probe detection (labResult: Positive) - 96119-3 SARS-CoV-2 (COVID-19) Ag [Presence] in Upper respiratory specimen by Immunoassay (labResult: Positive) - 94558-4 SARS-CoV-2 (COVID-19) Ag [Presence] in Respiratory specimen by Rapid immunoassay (labResult: Positive) | **Exclude hospitalization from three days before to two days after COVID-19 diagnosis**  **Hospitalization**   - CPT:1013659 Hospital Inpatient Services - Visit: Inpatient Encounter - Visit: Short Stay - Visit: Inpatient Non-acute - CPT:99221 Initial hospital care, per day, for the evaluation and management of a patient, which requires these 3 key components: A detailed or comprehensive history; A detailed or comprehensive examination; and Medical decision making that is straightforward or of low complexity. Counseling and/or coordination of care with other physicians, other qualified health care professionals, or agencies are provided consistent with the nature of the problem(s) and the patient's and/or family's needs. Usually, the problem(s) requiring admission are of low severity. Typically, 30 minutes are spent at the bedside and on the patient's hospital floor or unit. - CPT:99222 Initial hospital care, per day, for the evaluation and management of a patient, which requires these 3 key components: A comprehensive history; A comprehensive examination; and Medical decision making of moderate complexity. Counseling and/or coordination of care with other physicians, other qualified health care professionals, or agencies are provided consistent with the nature of the problem(s) and the patient's and/or family's needs. Usually, the problem(s) requiring admission are of moderate severity. Typically, 50 minutes are spent at the bedside and on the patient's hospital floor or unit. - CPT:99223 Initial hospital care, per day, for the evaluation and management of a patient, which requires these 3 key components: A comprehensive history; A comprehensive examination; and Medical decision making of high complexity. Counseling and/or coordination of care with other physicians, other qualified health care professionals, or agencies are provided consistent with the nature of the problem(s) and the patient's and/or family's needs. Usually, the problem(s) requiring admission are of high severity. Typically, 70 minutes are spent at the bedside and on the patient's hospital floor or unit. - CPT:99231 Subsequent hospital care, per day, for the evaluation and management of a patient, which requires at least 2 of these 3 key components: A problem focused interval history; A problem focused examination; Medical decision making that is straightforward or of low complexity. Counseling and/or coordination of care with other physicians, other qualified health care professionals, or agencies are provided consistent with the nature of the problem(s) and the patient's and/or family's needs. Usually, the patient is stable, recovering or improving. Typically, 15 minutes are spent at the bedside and on the patient's hospital floor or unit. - CPT:99232 Subsequent hospital care, per day, for the evaluation and management of a patient, which requires at least 2 of these 3 key components: An expanded problem focused interval history; An expanded problem focused examination; Medical decision making of moderate complexity. Counseling and/or coordination of care with other physicians, other qualified health care professionals, or agencies are provided consistent with the nature of the problem(s) and the patient's and/or family's needs. Usually, the patient is responding inadequately to therapy or has developed a minor complication. Typically, 25 minutes are spent at the bedside and on the patient's hospital floor or unit. - CPT:99233 Subsequent hospital care, per day, for the evaluation and management of a patient, which requires at least 2 of these 3 key components: A detailed interval history; A detailed examination; Medical decision making of high complexity. Counseling and/or coordination of care with other physicians, other qualified health care professionals, or agencies are provided consistent with the nature of the problem(s) and the patient's and/or family's needs. Usually, the patient is unstable or has developed a significant complication or a significant new problem. Typically, 35 minutes are spent at the bedside and on the patient's hospital floor or unit. - CPT:99234 Observation or inpatient hospital care, for the evaluation and management of a patient including admission and discharge on the same date, which requires these 3 key components: A detailed or comprehensive history; A detailed or comprehensive examination; and Medical decision making that is straightforward or of low complexity. Counseling and/or coordination of care with other physicians, other qualified health care professionals, or agencies are provided consistent with the nature of the problem(s) and the patient's and/or family's needs. Usually the presenting problem(s) requiring admission are of low severity. Typically, 40 minutes are spent at the bedside and on the patient's hospital floor or unit. - CPT:99235 Observation or inpatient hospital care, for the evaluation and management of a patient including admission and discharge on the same date, which requires these 3 key components: A comprehensive history; A comprehensive examination; and Medical decision making of moderate complexity. Counseling and/or coordination of care with other physicians, other qualified health care professionals, or agencies are provided consistent with the nature of the problem(s) and the patient's and/or family's needs. Usually the presenting problem(s) requiring admission are of moderate severity. Typically, 50 minutes are spent at the bedside and on the patient's hospital floor or unit. - CPT:99236 Observation or inpatient hospital care, for the evaluation and management of a patient including admission and discharge on the same date, which requires these 3 key components: A comprehensive history; A comprehensive examination; and Medical decision making of high complexity. Counseling and/or coordination of care with other physicians, other qualified health care professionals, or agencies are provided consistent with the nature of the problem(s) and the patient's and/or family's needs. Usually the presenting problem(s) requiring admission are of high severity. Typically, 55 minutes are spent at the bedside and on the patient's hospital floor or unit. - CPT:99238 Hospital discharge day management; 30 minutes or less - CPT:99239 Hospital discharge day management; more than 30 minutes - CPT:99251 Inpatient consultation for a new or established patient, which requires these 3 key components: A problem focused history; A problem focused examination; and Straightforward medical decision making. Counseling and/or coordination of care with other physicians, other qualified health care professionals, or agencies are provided consistent with the nature of the problem(s) and the patient's and/or family's needs. Usually, the presenting problem(s) are self-limited or minor. Typically, 20 minutes are spent at the bedside and on the patient's hospital floor or unit. - CPT:99252 Inpatient consultation for a new or established patient, which requires these 3 key components: An expanded problem focused history; An expanded problem focused examination; and Straightforward medical decision making. Counseling and/or coordination of care with other physicians, other qualified health care professionals, or agencies are provided consistent with the nature of the problem(s) and the patient's and/or family's needs. Usually, the presenting problem(s) are of low severity. Typically, 40 minutes are spent at the bedside and on the patient's hospital floor or unit. - CPT:99254 Inpatient consultation for a new or established patient, which requires these 3 key components: A comprehensive history; A comprehensive examination; and Medical decision making of moderate complexity. Counseling and/or coordination of care with other physicians, other qualified health care professionals, or agencies are provided consistent with the nature of the problem(s) and the patient's and/or family's needs. Usually, the presenting problem(s) are of moderate to high severity. Typically, 80 minutes are spent at the bedside and on the patient's hospital floor or unit. - CPT:99255 Inpatient consultation for a new or established patient, which requires these 3 key components: A comprehensive history; A comprehensive examination; and Medical decision making of high complexity. Counseling and/or coordination of care with other physicians, other qualified health care professionals, or agencies are provided consistent with the nature of the problem(s) and the patient's and/or family's needs. Usually, the presenting problem(s) are of moderate to high severity. Typically, 110 minutes are spent at the bedside and on the patient's hospital floor or unit. - CPT:99253 Inpatient consultation for a new or established patient, which requires these 3 key components: A detailed history; A detailed examination; and Medical decision making of low complexity. Counseling and/or coordination of care with other physicians, other qualified health care professionals, or agencies are provided consistent with the nature of the problem(s) and the patient's and/or family's needs. Usually, the presenting problem(s) are of moderate severity. Typically, 55 minutes are spent at the bedside and on the patient's hospital floor or unit. |
| Patients with NAFLD | - K76.0 Fatty (change of) liver, not elsewhere classified - K75.81 Nonalcoholic steatohepatitis (NASH) | **Chronic liver diseases not related to NAFLD**   - K72 Hepatic failure, not elsewhere classified - K76.2 Central hemorrhagic necrosis of liver - K70.0 Alcoholic fatty liver - K70.10 Alcoholic hepatitis without ascites - K70.30 Alcoholic cirrhosis of liver without ascites - K70.9 Alcoholic liver disease, unspecified - K73.0 Chronic persistent hepatitis, not elsewhere classified - K73.2 Chronic active hepatitis, not elsewhere classified - K73.8 Other chronic hepatitis, not elsewhere classified - K73.9 Chronic hepatitis, unspecified - K74.0 Hepatic fibrosis - K74.3 Primary biliary cirrhosis - K74.4 Secondary biliary cirrhosis - K74.5 Biliary cirrhosis, unspecified - K75.4 Autoimmune hepatitis - K76.7 Hepatorenal syndrome - K77 Liver disorders in diseases classified elsewhere - K71.6 Toxic liver disease with hepatitis, not elsewhere classified - B17 Other acute viral hepatitis - B17.10 Acute hepatitis C without hepatic coma - B17.2 Acute hepatitis E - B17.8 Other specified acute viral hepatitis - B18.2 Chronic viral hepatitis C - B18.8 Other chronic viral hepatitis - B18.9 Chronic viral hepatitis, unspecified - B00.81 Herpesviral hepatitis - B15.0 Hepatitis A with hepatic coma - B15.9 Hepatitis A without hepatic coma - B16.0 Acute hepatitis B with delta-agent with hepatic coma - B16.1 Acute hepatitis B with delta-agent without hepatic coma - B16.2 Acute hepatitis B without delta-agent with hepatic coma - B16.9 Acute hepatitis B without delta-agent and without hepatic coma - B17.11 Acute hepatitis C with hepatic coma - B17.2 Acute hepatitis E - B17.8 Other specified acute viral hepatitis - B17.9 Acute viral hepatitis, unspecified - B18.0 Chronic viral hepatitis B with delta-agent - B18.1 Chronic viral hepatitis B without delta-agent - B18.2 Chronic viral hepatitis C - B19.0 Unspecified viral hepatitis with hepatic coma - B19.11 Unspecified viral hepatitis B with hepatic coma - B19.2 Unspecified viral hepatitis C - B19.21 Unspecified viral hepatitis C with hepatic coma - B19.9 Unspecified viral hepatitis without hepatic coma - B25.1 Cytomegaloviral hepatitis - B26.81 Mumps hepatitis - B58.1 Toxoplasma hepatitis - B26.81 Mumps hepatitis - B58.1 Toxoplasma hepatitis - B94.2 Sequelae of viral hepatitis - K70.11 Alcoholic hepatitis with ascites - E83.01 Wilson's disease - K76.9 Liver disease, unspecified - K72.9 Hepatic failure, unspecified - K73.1 Chronic lobular hepatitis, not elsewhere classified - K72.90 Hepatic failure, unspecified without coma - K73.1 Chronic lobular hepatitis, not elsewhere classified - K75.2 Nonspecific reactive hepatitis - K75.3 Granulomatous hepatitis, not elsewhere classified - K75.89 Other specified inflammatory liver diseases - K70.31 Alcoholic cirrhosis of liver with ascites - F10.2 Alcohol dependence - K71.7 Toxic liver disease with fibrosis and cirrhosis of liver |
| Patients with antiviral agents | - RXNORM:2587901 molnupiravir - RXNORM:2587892 nirmatrelvir - RXNORM:85762 ritonavir   (Exclude the above criteria when applying the control group) | - HCPCS M0220 Injection, tixagevimab and cilgavimab, for the pre-exposure prophylaxis only, for certain adults and pediatric individuals (12 years of age and older weighing at least 40kg) with no known sars-cov-2 exposure, who either have moderate to severely compromised immune systems or for whom vaccination with any available covid-19 vaccine is not recommended due to a history of severe adverse reaction to a covid-19 vaccine(s) and/or covid-19 vaccine component(s), includes injection and post administration monitoring - HCPCS Q0220 Injection, tixagevimab and cilgavimab, for the pre-exposure prophylaxis only, for certain adults and pediatric individuals (12 years of age and older weighing at least 40kg) with no known sars-cov-2 exposure, who either have moderate to severely compromised immune systems or for whom vaccination with any available covid-19 vaccine is not recommended due to a history of severe adverse reaction to a covid-19 vaccine(s) and/or covid-19 vaccine component(s), 300 mg - HCPCS M0222 Intravenous injection, bebtelovimab, includes injection and post administration monitoring - HCPCS XW13325 Transfusion of Convalescent Plasma (Nonautologous) into Peripheral Vein, Percutaneous Approach, New Technology Group 5 - HCPCS XW14325 Transfusion of Convalescent Plasma (Nonautologous) into Central Vein, Percutaneous Approach, New Technology Group 5 - HCPCS Q0222 Injection, bebtelovimab, 175 mg - RXNORM 2284718 remdesivir - RXNORM 2587300 Tixagevimab - RXNORM 2592360 Bebtelovimab - RXNORM 2587306 Cilgavimab |
